# Eprenetapopt in combination with carboplatin in high-grade ovarian and triple negative breast cancer cell lines with acquired resistance to olaparib

**DOI:** 10.3389/fonc.2026.1754873

**Published:** 2026-04-02

**Authors:** Begoña Pineda, Juan Jose Martinez-Pretel, Paloma Sanchez- Serrano, Stergios Boussios, Maria Rodrigo-Faus, Martín Pérez-Leal, Elena Obrador, Javier Perea, J. Alejandro Perez-Fidalgo

**Affiliations:** 1 Department of Physiology, Faculty of Medicine and Odontology, University of Valencia, Valencia, Spain; 2 Biomedical Research Institute (INCLIVA), Valencia, Spain; 3 Faculty of Medicine, School of Health Sciences, University of Ioannina, Ioannina, Greece; 4 Department of Medical Oncology, University Hospital of Ioannina, Ioannina, Greece; 5 Faculty of Health Sciences, Universidad Europea de Valencia, Valencia, Spain; 6 Centro Avanzado de Microbiología Aplicada, Universitat Politècnica de València, Valencia, Spain; 7 Department of Medical Oncology, Hospital Clinico Universitario de Valencia, Valencia, Spain; 8 Department of Medicine, University of Valencia, Valencia, Spain; 9 CIBERONC, Madrid, Spain

**Keywords:** carboplatin, eprenetapopt, high grade serous ovarian cancer, olaparib, synergism, triple negative breast cancer

## Abstract

**Background:**

High-grade serous ovarian cancer (HGSOC) and triple-negative breast cancer (TNBC) frequently exhibit mutations in DNA damage response (DDR) genes, as *BRCA1, BRCA2* and *TP53*, which are associated with chemotherapy sensitivity. Olaparib, a PARP inhibitor, provides the greatest clinical benefit as maintenance therapy in HGSOC—particularly in tumors with BRCA1/2 mutations or broader homologous recombination deficiency (HRD)—whereas benefit in HRD−negative disease is limited. Eprenetapopt (APR-246) restores wild-type p53 function in tumor cells with *TP53* mutations. This study investigates the potential of combining eprenetapopt with carboplatin to overcome resistance to the PARP inhibitor (PARPi) olaparib in HGSOC and TNBC cell lines.

**Methods:**

As preclinical models, human ovarian cancer cell lines (HGSOC) (PEO1, Kuramochi) and a triple-negative breast cancer cell line (MDA-MB-231) were utilized as parental lines to generate their respective olaparib-resistant cell lines (PEO1R, Kuramochi-R, MDA-MB-231-R) by exposing the cells to increasing doses of olaparib. Cell viability, apoptosis and cell cycle progression, were assessed using MTT assays and Annexin V assay and propidium iodide staining, respectively. The Chou-Talalay method was used to calculate the combination index (CI) for drug synergism.

**Results:**

Olaparib-resistance was confirmed in both HGSOC and TNBC cell lines, as they exhibited significantly higher IC_50_ values compared to their respective parental lines. Moreover, the olaparib-resistant cells exhibited cross-resistance to carboplatin. Remarkably, the combination of eprenetapopt with carboplatin showed a synergistic effect in both parental and resistant cell lines, reducing tumor cell viability and demonstrating synergistic interaction compared with single agents. Combinatorial treatment also significantly increased apoptotic cell population in parental HGSOC and TNBC cell lines as well as in MDA-MB-231-R olaparib-resistant cells. Cell cycle analysis revealed that carboplatin mainly induced a significant increase in S or G2/M phase accumulation and a reduction in G1-phase cells, with eprenetapopt having a minimal additional impact.

**Conclusions:**

Combining eprenetapopt with carboplatin shows promising preclinical efficacy by enhancing cytotoxicity in olaparib−resistant models and demonstrating synergistic interaction; these data support the combination as a potential strategy to mitigate PARPi resistance and carboplatin cross−resistance in *TP53* mutant HGSOC and TNBC cell lines. Although further studies are needed to elucidate the molecular mechanisms underlying the synergistic effect, here we point out the combination of eprenetapopt and carboplatin as a potential therapeutic strategy to address olaparib resistance in HGSOC and TNBC patients.

## Background

High grade serous ovarian cancer (HGSOC) is an aggressive histologic subtype of ovarian cancer in which *TP53* mutation represent one of the most common genetic alterations. In fact, *TP53* mutations are considered present in up to 96% of HGSOC cases (1). Similarly, in triple negative breast cancer (TNBC) the prevalence of *TP53* mutations is approximately 60% (2, 3).

PARP inhibitors (PARPi) such as olaparib, a family of agents targeting DNA damage response (DDR) mechanisms, have shown an important benefit in these tumors, particularly in *BRCA*-mutated patients in both HGSOC and TNBC (4). Olaparib inhibits PARP1/2 and also “traps” PARP−DNA complexes at single−strand break sites, which can stall or collapse replication forks and generate lethal double−strand breaks. In HRD/BRCA−deficient tumors, this leads to synthetic lethality (4). In newly diagnosed HGSOC, PARP inhibitors are used primarily as maintenance therapy after response to platinum chemotherapy (as monotherapy or combined with bevacizumab), with greatest benefit in BRCA−mutated and HRD−positive tumors (5–7). However also BRCA wild type (wt) patients will benefit from PARPi and the maintenance treatment after platinum is also recommended (8). Unfortunately, many patients will progress after PARPi maintenance and therefore, resistance to PARPi is becoming a frequent situation in the clinics. Beyond BRCA reversion events, PARPi resistance can also arise through restoration of homologous recombination (e.g., loss of 53BP1/Shieldin or true BRCA reversions), replication−fork stabilization, alterations that reduce PARP−DNA trapping, and increased drug efflux.

P53 is a protein involved in cell cycle regulation by arresting the cycle in G1 and regulating apoptosis and cell death initiation. Accordingly, TP53 encodes a transcription factor that orchestrates DNA−damage responses and cell−cycle checkpoints and is frequently altered in HGSOC and TNBC (9). Consequently, mutations in *TP53* are closely related to carcinogenesis and act as key drivers of cancer in several solid tumors. Moreover, *TP53* mutations have been associated with a higher sensitivity to chemotherapy, specifically platinum-based compounds. However, mutational diversity suggests that not all mutations are equivalent and that alterations in the specific protein domains may have different effects, including variations in chemotherapy sensitivity (10).

Eprenetapopt (also known as APR-246 or PRIMA-1^MET^) is a first-in-class *TP53* modulator that, through binding to the cysteine residues in the mutated p53 protein, restores its wt conformation and its function in tumor cells carrying *TP53* mutations (11). Sensitivity to eprenetapopt can vary with the specific TP53 mutation class and with redox/cystine–glutathione context.

Preclinical studies confirmed that eprenetapopt was able to overcome cisplatin and doxorubicin resistance in ovarian cancer cells (12). Nevertheless, the mechanistic insights into this compound’s activity, particularly in combination with carboplatin, remain largely unexplored in the context of tumor cells exhibiting resistance to PARPi such as olaparib.

In the PISARRO study, a phase 1b clinical trial exploring safety and tolerability of the combination of carboplatin and pegylated liposomal doxorubicin (PLD) with eprenetapopt, the most relevant toxicity attributable to eprenetapopt was dizziness with 71% of patients presenting this adverse effect, although most was grade 1−2 (13). The combination was considered tolerable and dose limiting toxicities (DLTs) such as myelosuppression and fatigue, were mainly related with chemotherapy. The activity of carboplatin + PLD + eprenetapopt shown in the PISARRO study was encouraging in the platinum−sensitive ovarian cancer setting14. However, this study was performed in pre−PARPi era and none of the enrolled patients were progressing after PARPi therapy (13).

Consequently, there is an urgent need to identify mechanisms of resistance to PARPi and strategies to address this acquired resistance. Therefore, the objective of our study was to generate olaparib−resistant cellular models of HGSOC and TNBC and to evaluate whether combining eprenetapopt with carboplatin enhances cytotoxicity and yields synergistic interactions in these resistant models. Secondary objectives included the assessment of cell−cycle progression, and apoptosis after eprenetapopt, carboplatin, or their combination. However, whether carboplatin + eprenetapopt improves cytotoxicity and demonstrates synergy specifically in olaparib−resistant, TP53−mutant HGSOC/TNBC had not been systematically tested prior to this study.

Our results demonstrate that combining eprenetapopt with carboplatin yields synergy and enhances cytotoxicity in Kuramochi, PEO1, and MDA−MB−231 models, including their olaparib−resistant counterparts.

## Methods

### Cancer cell lines and reagents

The human ovarian cancer cell line Kuramochi was obtained from the Japanese Collection of Research Bioresoures Cell Bank (#JCRB0098, JCBR). The human ovarian cancer cell line PEO1 was kindly donated by Dr. Marta Mendiola laboratory at La Paz University Hospital (Madrid, Spain). The human triple negative breast cancer cell line MDA-MB-231 was obtained from the American Type Culture Collection (HTB-26, ATCC). All cell lines, Kuramochi, PEO1 and MDA-MB-231, were maintained in medium RPMI1640 (#L0500-500, Labclinics), DMEM High Glucose (#L0100-500, Labclinics) and DMEM F-12 (#L0090-500, Labclinics), respectively, supplemented with 10% Fetal Bovine Serum and 1% Penicillin-Streptomycin at 37 °C and 5% CO_2_.

Olaparib (S1060, Selleckchem) and Eprenetapopt (S7724, Selleckchem) were resuspended in dimethyl sulphoxide (DMSO) following the manufacturer’s instructions. Carboplatin (#664732.5, Pfizer) was kindly donated by the Pharmacy Department of the Clinic University Hospital (Valencia, Spain).

### Olaparib−resistant cell lines generation

The resistant cell lines MDA-MB-231-R and Kuramochi-R were established in our laboratory by exposing the parental cell lines to escalating doses of olaparib for 72-hour intervals, followed by recovery periods ranging from one week to one month before subsequent treatments. For the initial doses, concentrations below the IC_50_ were used (20 μM for MDA-MB-231 and Kuramochi) and once treatment doses exceeded the IC_50_, resistance was verified using an MTT assay comparing the IC_50_ for olaparib in the generated cell line with the IC_50_ of its parental cell line.

On the other hand, the olaparib-resistant cell line PEO1-R was kindly provided by the collaborating group of Dr. Marta Mendiola (Hospital Universitario La Paz, Madrid, Spain). This cell line was generated using a protocol slightly different from that employed in our laboratory. Specifically, the PEO1 cell line was exposed to an olaparib concentration exceeding the IC_50_ (20 μM), after which resistant cell clones were isolated and allowed sufficient time to recover before undergoing subsequent treatments. Once the treatment was no longer effective in the cell line, resistance was verified using an MTT assay, comparing the IC_50_ for olaparib in the generated cell line with the IC_50_ of its parental cell line. To maintain acquired resistance to olaparib, all cell lines were treated monthly with an olaparib dose corresponding to their parental IC_50_ for a period of 72h (100 μM for MDA-MB-231-R, 100 μM for Kuramochi-R and 20 μM for PEO1-R).

### *In vitro* cell viability assay

Cell viability was determined using the 3-(4,5-dimethylthiazol-2-yl)-2,5-diphenyltetrazolium bromide (MTT) Cell Proliferation Kit I (11465007001, Roche Applied Science), which measures the conversion of the yellow tetrazolium into purple formazan by mitochondrial redox activity in living cells. For this purpose, cells were seeded at a density of 5 x 10 (3) per well in 96-well plates. After 24h, cells were treated with increased concentrations of olaparib (5 μM to 500 μM for Kuramochi and Kuramochi-R and 2,5 μM to 300 μM for MDA-MB-231, MDA-MB-231-R, PEO1 and PEO1-R cells), carboplatin (5 μM to 500 μM in Kuramochi and Kuramochi-R cell lines, 50 μM to 3000 μM in MDA-MB-231 and MDA-MB-231-R and 10 μM to 160 μM in PEO1 and PEO1-R cells) and eprenetapopt (1 μM to 70 μM for Kuramochi, Kuramochi-R, MDA-MB-231 and MDA-MB-231-R cells and from 1 μM to 40 μM for PEO1 and PEO1-R cells) performing triplicates for each concentration. In each experiment, DMSO-treated cells were used as controls, except for the carboplatin treatment, which was prepared in PBS. The DMSO concentration applied to the cells corresponded to the highest concentration used in the treatment wells. After 72h of incubation at 37 °C and 5% CO_2_, cell media was removed and MTT solution (0.5 mg/mL) was added to each well. After incubating cells for 3–4 h at 37 °C and 5% CO_2_, the MTT solution was removed, and the purple formazan crystals were dissolved in isopropanol. The cellular viability was evaluated measuring the main absorbance at 570 nm and the nonspecific background absorbance at 690 nm by using a microplate spectrophotometer SpectraMax Plus (Molecular Devices, Thermo Fisher Scientific). The viability was calculated as the percentage of the mean absorbance of each triplicate divided by the mean absorbance of the control condition. The absorbance data obtained were processed using the GraphPad Prism program (version 8.02; GraphPad Software, Inc., La Jolla, CA, USA) to determine the half-maximal inhibitory concentration (IC_50_), defined as the drug concentration required to reduce cell viability by 50% compared to untreated cells.

### Mutational status analysis of *TP53*, *BRCA1* and *BRCA2* genes

To verify mutational pattern for *TP53*, *BRCA1* and *BRCA2* genes, a comparative sequencing analysis was performed with the sensitive cell lines (Kuramochi, PEO1 and MDA-MB-231) and the olaparib-resistant cell lines (Kuramochi-R, PEO1-R and MDA-MB-231-R). This analysis was carried out by the Precision Medicine Unit of IIS INCLIVA. First, DNA extraction was performed using automated purification with paramagnetic beads on the Maxwell 16 LEV system (Promega, Madrid, Spain) with the Maxwell^®^ 16 LEV Blood DNA Kit (Promega), following the manufacturer’s instructions. Subsequently, the exonic regions of the *TP53*, *BRCA1* and *BRCA2* genes were amplified using panels designed by the Precision Medicine Unit of IIS INCLIVA. These genes were profiled because alterations in TP53 and BRCA1/2 commonly contribute to DDR defects and tumor biology in HGSOC/TNBC models.

Library generation was carried out through a two-step polymerase chain reaction (PCR) using the Qiagen Multiplex enzyme and Nextera adapters (Illumina) as sequencing adapters. Finally, sequencing was performed on Illumina platforms with a 150PE configuration, ensuring a minimum coverage of 250X.

### Eprenetapopt and carboplatin synergism analysis

To study the synergism between eprenetapopt and carboplatin, the combination index (CI) in all the cell lines was determined using the Chou-Talalay assay (14, 15). Cells were treated for 72 hours at 37 °C and 5% CO_2_. Single-agent dose–response curves were generated using eprenetapopt at concentrations ranging from 0.5 to 200 µM (0-150 µM for MDA-MB-231, 0-70 µM for MDA-MB-231-R, 0-150 µM for Kuramochi, 0-200 µM for Kuramochi-R, 0-40 µM for PEO1 and 0-30 µM for PEO1-R) and carboplatin from 1 to 1820 µM (0-825 µM for MDA-MB-231, 0-1820 µM for MDA-MB-231-R, 0-225 µM for Kuramochi, 0-240 µM for Kuramochi-R, 0-160 µM for PEO1 and 0-123 µM for PEO1-R).

For combination experiments, drugs were combined at a fixed-dose ratio to generate dose–response matrices for Chou–Talalay analysis. The fixed-dose ratio of eprenetapopt:carboplatin was defined based on the concentration ranges used in the single-agent dose–response viability curves and determined independently for each cell line. Proportions of the two drugs were maintained constant across the combination dose–response matrices used for Chou–Talalay synergy analysis. The resulting fixed-dose ratios were: 1:5.5 for MDA-MB-231, 1:26 for MDA-MB-231-R, 1:1.5 for Kuramochi, 1:1.875 for Kuramochi-R, 1:4 for PEO1 and 1:7 for PEO1-R.

Each experiment was performed in at least 3 independent biological replicates, with ≥3 technical replicates per point. The results about the cell viability were determined using MTT assay. Once the viability data were processed, they were analyzed using the Compusyn software (Biosoft) (14, 15,)which calculates the combination index (CI) values corresponding to the fraction affected by the dose (Fa) computed as 1 − (normalized viability), specifically those related to IC25 (Fa = 0.25), IC50 (Fa = 0.50), and IC75 (Fa = 0.75) for the drug combination. A CI greater than 1 indicates antagonism, equal to 1 an additive effect, and less than 1 synergism. The exact drug concentrations used in each combination matrix and the dosing scheme used to generate CI values for each cell line are provided in the CompuSyn reports deposited in the public repository (see Data Availability section).

### Apoptosis analysis

To assess changes in apoptosis levels between untreated and treated cells with eprenetapopt and/or carboplatin, in both parental and olaparib-resistant cell lines, an apoptosis assay using flow cytometry was performed. All cell lines, after reaching 70-80% confluence, were treated with the treatments eprenetapopt (APR), carboplatin (CBP), or the combination (COMBO), or without treatment (CNT), for a period of 72h at 37°C and 5% CO_2_. After this time, 150.000 cells from each condition were resuspended in 100 μL of 1X Annexin V binding buffer (#BB10X, Inmunostep) in distilled water. Subsequently, 5 μL of Annexin V (#ANXVF-200T, Inmunostep) was added to each sample, allowing the detection of apoptotic cells due to its ability to bind to phosphatidylserine, which serves as an apoptosis marker when located on the outer cell membrane. The samples were incubated for 10 minutes in the dark at room temperature. After this incubation, 2.5 μL of Propidium Iodide (PI) (#P4170, Sigma-Aldrich) was added to each sample to detect cells with compromised membrane integrity, as only these cells can take up the dye. The samples were incubated for an additional 5 minutes in the dark at room temperature. Unstained cells as well as single-stained controls (Annexin V–FITC only and PI only) were used to set voltages, define background fluorescence, and perform fluorescence compensation. A sequential gating strategy was applied to exclude debris based on forward and side scatter parameters and to remove doublets using FSC-A versus FSC-H plots. Quadrant gates were defined based on negative controls, allowing identification of viable cells (Q3, Annexin V−/PI−), early apoptotic cells (Q4, Annexin V+/PI−), late apoptotic and necrotic cells (Q2, Annexin V+/PI+), and necrotic cells (Q1, Annexin V−/PI+). At least 10,000 events per sample were analyzed. Flow cytometry analyses were performed with BDFostessa X-20 cytometer and BD FACSDiva software (v8.0.1) for analysis. All experiments were performed with three independent biological replicates.

### Cell cycle analysis by flow cytometry

All cell lines, after eprenetapopt (APR), carboplatin (CBP) or combination treatment (COMBO) for 72h or without treatment (CNT), were trypsinized, washed in PBS and fixed in 70% ethanol at −20 °C overnight. Ethanol was removed by centrifugation at 1.500 rpm for 5 min and cells were stained with propidium iodide (50 μg/mL) in the presence of RNase A (50 μg/mL) for 24h at 4 °C. The BD LSRFortessa™ X-20 Cell Analyzer (BD biosciences) was used and the data obtained was analyzed using FlowJo™ v10.8 Software (BD Life Sciences) (16). DNA content histograms were modeled in FlowJo v10.8 using the Watson (Pragmatic) model. Debris was excluded by FSC/SSC gating; doublets/aggregates were removed using PI−Area vs. PI−Width (and PI−Height) plots prior to model fitting. A minimum of 20,000 singlet events per sample was required for analysis.

### Statistical analysis

Statistical analyses were performed using GraphPad Prism version 8.0.2 for Windows, GraphPad Software, Boston, Massachusetts USA, https://www.graphpad.com. First, for the viability MTT assays of olaparib and carboplatin the IC_50_ values of drugs were determined through a nonlinear regression using the transformation X = log(X), where X represents drug concentration, and the corresponding formula was applied:


$$Y=\frac{100}{1+10^{\log\left(IC50-X\right)*slope}}$$

95% confidence intervals (CIs) for IC_50_ values were calculated using the standard error of the logIC_50_ obtained from the regression model in GraphPad Prism. Comparisons of IC50 between parental and resistant lines were performed using extra sum−of−squares F−tests (‘comparison of fits’, GraphPad Prism), evaluating a model with shared vs. separate logIC50 parameters. Significance was set at α=0.05.

For apoptosis and cell−cycle endpoints, data (n=3 independent experiments) were analyzed by one−way ANOVA followed by Tukey’s post−hoc test to control the family−wise error rate across all pairwise comparisons. Normality (Shapiro–Wilk) was assessed; if assumptions were not met, non−parametric tests were used.

## Results

### Generation of olaparib-resistant cell lines

Olaparib-resistant cell lines were derived from the parental MDA-MB-231, PEO1 and Kuramochi cell lines. They were generated by different methods, as was described in Material and methods section, to investigate whether the combination of eprenetapopt + carboplatin was also effective in the context of olaparib resistance.

**Figure 1:**
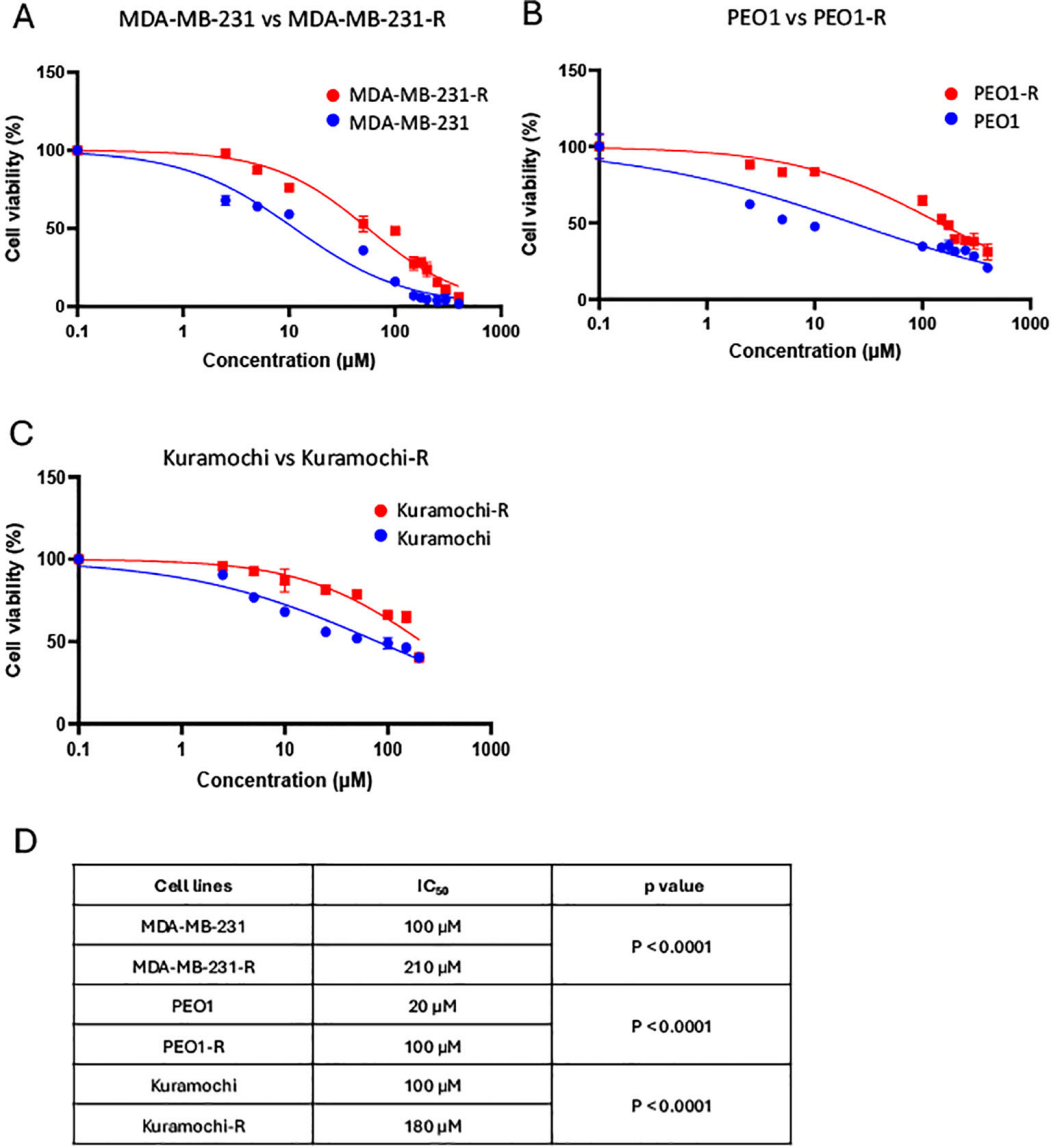
Increased dose exposure to olaparib induces acquired resistance in TNBC and HGSOC cell lines. **(A)** Viability assay of MDA-MB-231 (blue) versus MDA-MB-231-R (red) upon exposure to increasing doses of olaparib for 72h. **(B)** Viability assay of PEO1 (blue) versus PEO1-R (red) upon exposure to increasing doses of olaparib for 72h. **(C)** Viability assay of Kuramochi (blue) versus Kuramochi-R (red) upon exposure to increasing doses of olaparib for 72h. Each point represents mean ± SEM of n = 3 biological replicates with at least three technical replicates for each point. All conditions were analyzed by a nonlinear regression model and p value below 0.05 was considered statistically significant. **(D)** IC50 values in sensitive vs resistance lines.

To confirm the acquisition of resistance, an MTT viability assay was conducted. Both parental and resistant cell lines were treated for 72h with escalating concentrations of olaparib and the IC_50_ values obtained were compared. As illustrated in **Figure 1**, the results revealed statistically significant differences in cell survival between parental and resistant cell lines at higher doses of olaparib. Specifically, the MDA-MB-231 parental cell line exhibited an IC_50_ of 100 µM, whereas its resistant counterpart (MDA-MB-231-R) demonstrated an IC_50_ of 210 µM (p< 0.0001) (**Figure 1A**). Similarly, a statistically significant difference was observed between PEO1 parental sensitive cell line and its olaparib-resistant PEO1-R counterpart, with IC_50_ values of 20 µM and 100 µM, respectively (p< 0.0001) (**Figure 1B**). Likewise, olaparib-resistance was confirmed in the Kuramochi cell line, where the IC_50_ increased from 100 µM in the parental cells to 180 µM in the resistant counterpart (Kuramochi-R) (p< 0.0001) (**Figure 1C**). **Figure 1D** summarizes the IC_50_ for olaparib treatments in all cell lines and the p-values for the comparison between parental sensitive cell lines with their respective olaparib-resistant cell line. All these results show that the significant differences in IC_50_ values observed between the parental and resistant cell lines confirm that progressive exposure to olaparib effectively induced resistance in these models.

### Sequencing of *TP53* and *BRCA* mutations in olaparib-sensitive and resistant cell lines

To characterize the most frequent mutated genes in the HGSOC and TNBC cell lines, both in the parental and olaparib-resistant cell lines, exonic regions of *TP53*, *BRCA1* and *BRCA2* genes were sequenced by NGS. As shown in **Table 1**, no BRCA1 mutations were detected in either the parental cell lines or their respective resistant counterparts. In the case of *BRCA2*, different mutations were detected in PEO1 and Kuramochi parental cell lines and they were also maintained in their respective resistant cell lines PEO1-R and Kuramochi-R. The fact that the mutations observed in the parental cells are also maintained in their olaparib-resistant lines suggests that the mechanisms by which olaparib resistance is developed do not involve the reversion of *BRCA1* nor *BRCA2* mutations, so additional mechanisms must exist that mediate the acquisition of this resistance.

**Table 1:** Presence of *TP53* mutations in HGSOC and TNBC olaparib-resistant cell lines makes them potential eprenetapopt therapeutic targets.

Cell line	BRCA1 mutation	BRCA2 mutation	TP53 mutation
MDA-MB-231	**-**	**-**	p. R280K c.839G>A (100%)
MDA-MB-231-R	**-**	**-**	p. R280K c.839G>A (100%)
PEO1	**-**	p. Y1655* c.4965C>G (59%)	p. G244D c.731G>A (100%)
PEO1-R	**-**	p. Y1655* c.4965C>G(67%)	p. G244D c.731G>A (100%)
Kuramochi	**-**	p. R2318* c.6952C>T (64%)	p. D281Y c.841G>T (100%)
Kuramochi-R	**-**	p. R2318* c.6952C>T (66%)	p. D281Y c.841G>T (100%)

Table showing the name of the cell line where *BRCA1*, *BRCA2* or *TP53* exonic regions were analyzed. In each cell is shown the single-nucleotide mutation detected in each cell line for each of the genes analyzed and in between the parenthesis is shown the percentage of cells (%) of that specific cell line where the mutation was detected.

Nevertheless, our results show that all three parental cell lines MDA-MB-231, PEO1 and Kuramochi cell lines, presented single-nucleotide mutations in *TP53* gene, although they did not harbor the same exact single mutation. Importantly, the mutation was not reversed in any of their olaparib-resistant cell lines, as the mutation was maintained in the 100% of the population of olaparib-resistant cells. These results highlight the presence of *TP53* mutations in these HGSOC and TNBC cell lines making them potentially suitable for treatment with eprenetapopt, which is involved in restoring p53 function in *TP53*-mutated cell lines, see **Table 2** for type of BRCA2 and TP53 mutations.

**Table 2:** NGS data of TP53 and BRCA2 mutations detected in HGSOC and TNBC olaparib-resistant cell lines.

Gene	BRCA2	BRCA2	TP53	TP53	TP53
coordinates	chr13:32339320	chr13:32346841	chr17:7673781	chr17:7673779	chr17:7674232
HGVSc	NM_000059.4::c.4965C>G	NM_000059.4:c.6952C>T	NM_000546.6:c.839G>A	NM_000546.6:c.841G>T	NM_000546.6:c.731G>A
HGVSp	p.(Tyr1655Ter)	p.(Arg2318Ter)	p.(Arg280Lys)	p.(Asp281Tyr)	p.(Gly244Asp)
variant classification	pathogenic	pathogenic	pathogenic	pathogenic/Likely pathogenic	pathogenic/Likely pathogenic
MDA-MB-231			100%		
MDA-MB-231-R			100%		
PEO1	59%				100%
PEO1-R	67%				100%
Kuramochi		64%		100%	
Kuramochi-R		66%		100%	

### Cross-resistance to carboplatin in Olaparib-resistant HGSOC and TNBC cell lines

**Figure 2:**
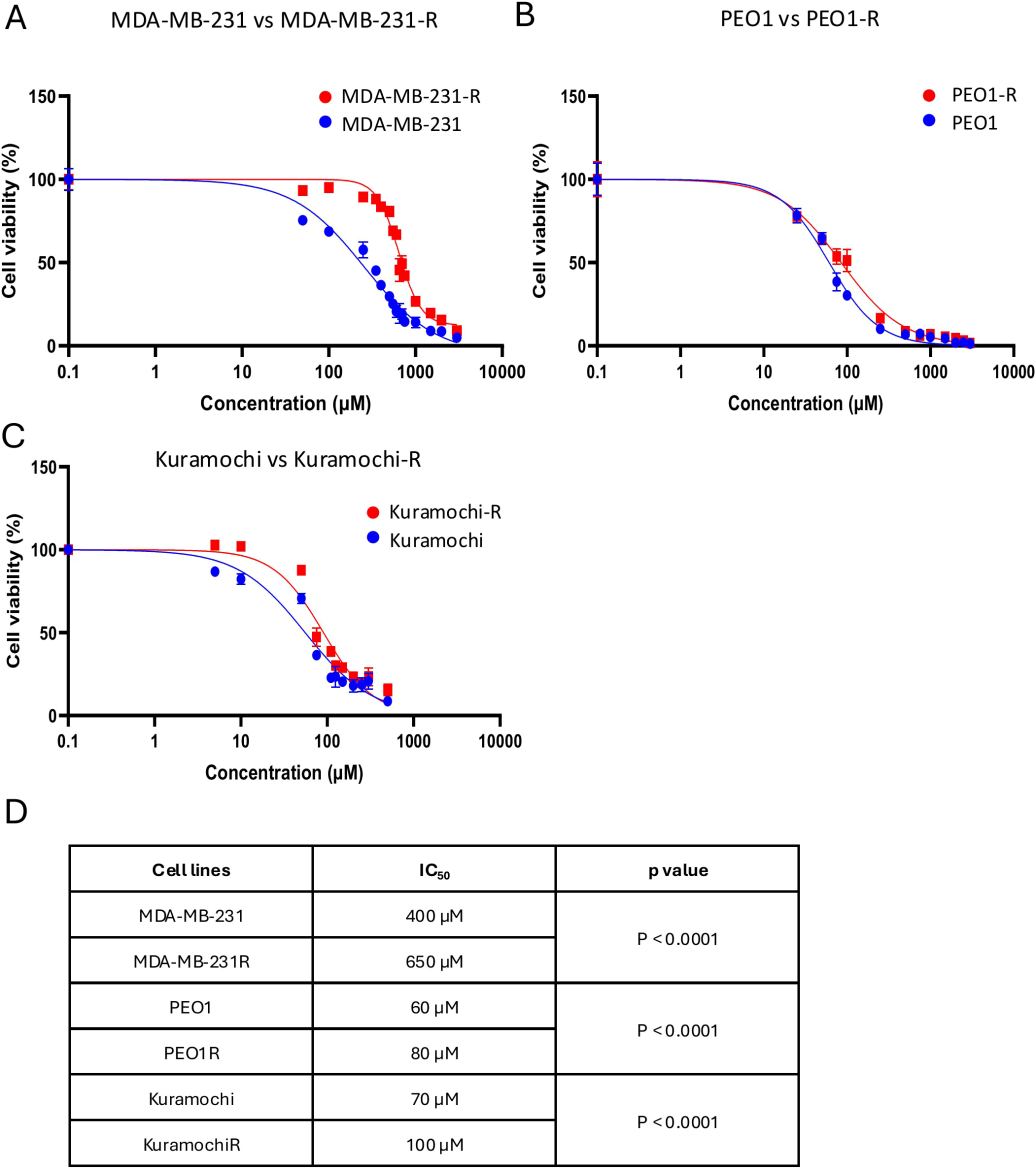
Olaparib-resistant TNBC and HGSOC cell lines show cross-resistance to carboplatin. **(A)** Viability assay of MDA-MB-231 (blue) versus MDA-MB-231-R (red) upon exposure to increasing doses of carboplatin for 72h. **(B)** Viability assay of PEO1 (blue) versus PEO1-R (red) upon exposure to increasing doses of carboplatin for 72h. **(C)** Viability assay of Kuramochi (blue) versus Kuramochi-R (red) upon exposure to increasing doses of carboplatin for 72h. Each point represents mean ± SEM of n = 3 biological replicates with at least three technical replicates for each point. All conditions were analyzed by a nonlinear regression model and p value below 0.05 was considered statistically significant. **(D)** IC50 values in sensitive vs resistance lines.

To determine whether olaparib-resistant cell lines also exhibited cross-resistance to carboplatin, we measured by an MTT viability assay the IC_50_ of parental and resistant cell lines following 72h of exposure to increasing carboplatin concentrations. Olaparib-resistant cell lines exhibited greater cell viability at higher carboplatin doses than their parental counterparts (see **Figure 2**). In the case of MDA-MB-231, the olaparib-resistant line (MDA-MB-231-R) displayed an IC_50_ of 650 µM for carboplatin, whereas the parental cells exhibited an IC_50_ of 400 µM (p< 0.0001) (**Figure 2A**). Although the differences between PEO1 and PEO1-R were less pronounced, they remained statistically significant, with IC_50_ values for carboplatin of 60 µM and 80 µM, respectively (p< 0.0001) (**Figure 2B**). Similarly, the olaparib-resistant Kuramochi-R cell line exhibited an IC_50_ of 100 µM, compared to 70 µM in the parental cells (p< 0.0001) (**Figure 2C**). **Figure 2D** summarizes the IC_50_ for carboplatin treatments in all cell lines and the p-values of the comparison of the parental sensitive cell lines with their respective olaparib-resistant cell line. Overall, these findings suggest that olaparib-resistant HGSOC and TNBC preclinical cell models generated in our laboratory exhibit increased tolerance to higher doses of carboplatin, indicating cross-resistance.

### Combination of carboplatin and eprenetapopt treatment shows a synergistic effect in the context of olaparib-resistance

**Figure 3:**
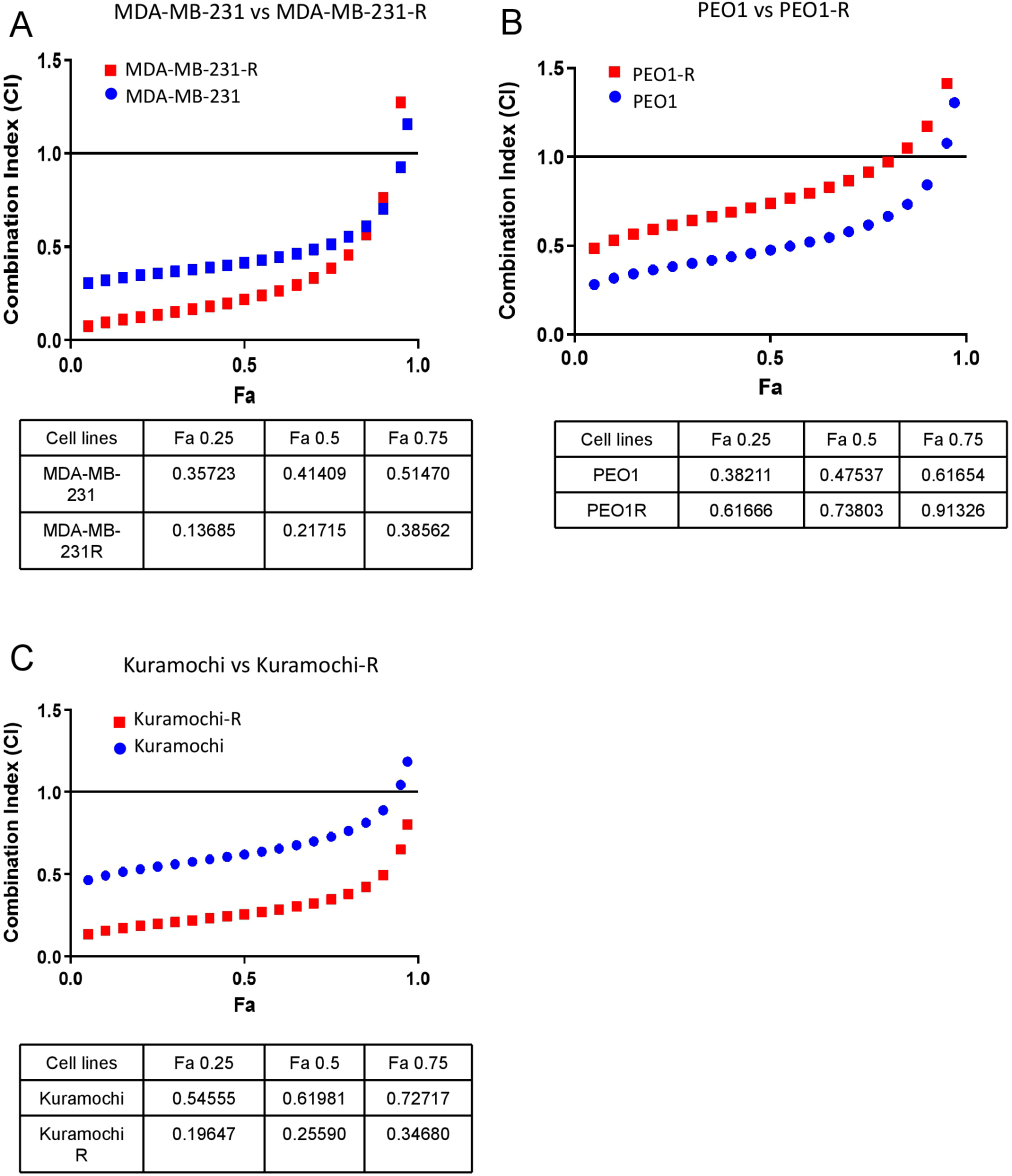
Chou−Talalay analyses demonstrate a synergistic interaction (CI<1) of carboplatin + eprenetapopt in both parental and resistant cell TNBC and HGSOC cell lines. **(A)** (Top) Dispersion graph showing the combination Index (CI) of Chou-Talalay synergism analyses in MDA-MB-231 (blue) versus MDA-MB-231-R (red) upon exposure to increasing doses of carboplatin + eprenetapopt for 72h and (bottom) table summarizing the main Chou-Talalay results **(B)** (Top) Dispersion graph showing the combination Index (CI) of Chou-Talalay synergism analyses in PEO1 (blue) versus PEO1-R (red) upon exposure to increasing doses of carboplatin + eprenetapopt for 72h and (bottom) table summarizing the main Chou-Talalay results. **(C)** (Top) Dispersion graph showing the combination Index (CI) of Chou-Talalay synergism analyses in Kuramochi (blue) versus Kuramochi-R (red) upon exposure to increasing doses of carboplatin + eprenetapopt for 72h and (bottom) table summarizing the main Chou-Talalay results.

To study if eprenetapopt could synergize with carboplatin reducing tumor cell survival even in the context of olaparib-resistance, we performed a Chou-Talalay (14, 15) analysis in both parental and olaparib-resistant cell lines. The Chou-Talalay algorithm calculates Combination Index (CI) values for specific fractions affected (Fa) by drug doses. A CI value less than 1 indicates synergy between the drugs, a value equal to 1 denotes an additive effect, and a value greater than 1 signifies antagonism. In our study, we focused our analyses on Fa values corresponding to IC_50_, as they reflect the efficacy of the combination of both drugs in reducing cell viability by 50%. The combination of carboplatin and eprenetapopt exhibited a synergistic effect in both MDA-MB-231 and MDA-MB-231-R cells, with CI at Fa = 0.5 of 0.41 and 0.22, respectively, indicating that eprenetapopt could enhance carboplatin efficacy even in olaparib-resistant settings (See **Figure 3**, **3A**). A synergistic effect was also observed in PEO1 and PEO1-R cells, with EO1 and PEO1−R, with CI at Fa = 0.5 of 0.48 and 0.74, respectively (**Figure 3B**). Likewise, the Kuramochi and Kuramochi-R cell lines displayed stronger synergism, particularly in the resistant line (CI at Fa = 0.5 of 0.62 and 0.26, respectively) (**Figure 3C**). Remarkably, in the case of Kuramochi-R and MDA-MB-231-R cell lines the synergistic effect observed between eprenetapopt and carboplatin was even higher than the one observed for their respective parental cell lines. These findings suggest that the combination of carboplatin + eprenetapopt represents a potential therapeutic alternative in HSGOC and TNBC patients with *TP53* mutations achieving a progression after olaparib treatment which could also potentially be effective, even in the context of cross-resistance to carboplatin taking into account that our olaparib-resistant cellular models demonstrated a cross-resistance to carboplatin.

### Eprenetapopt + carboplatin treatment shows a limited effect in increasing in apoptosis levels of TNBC and HGSOC olaparib-resistant cell lines

**Figure 4:**
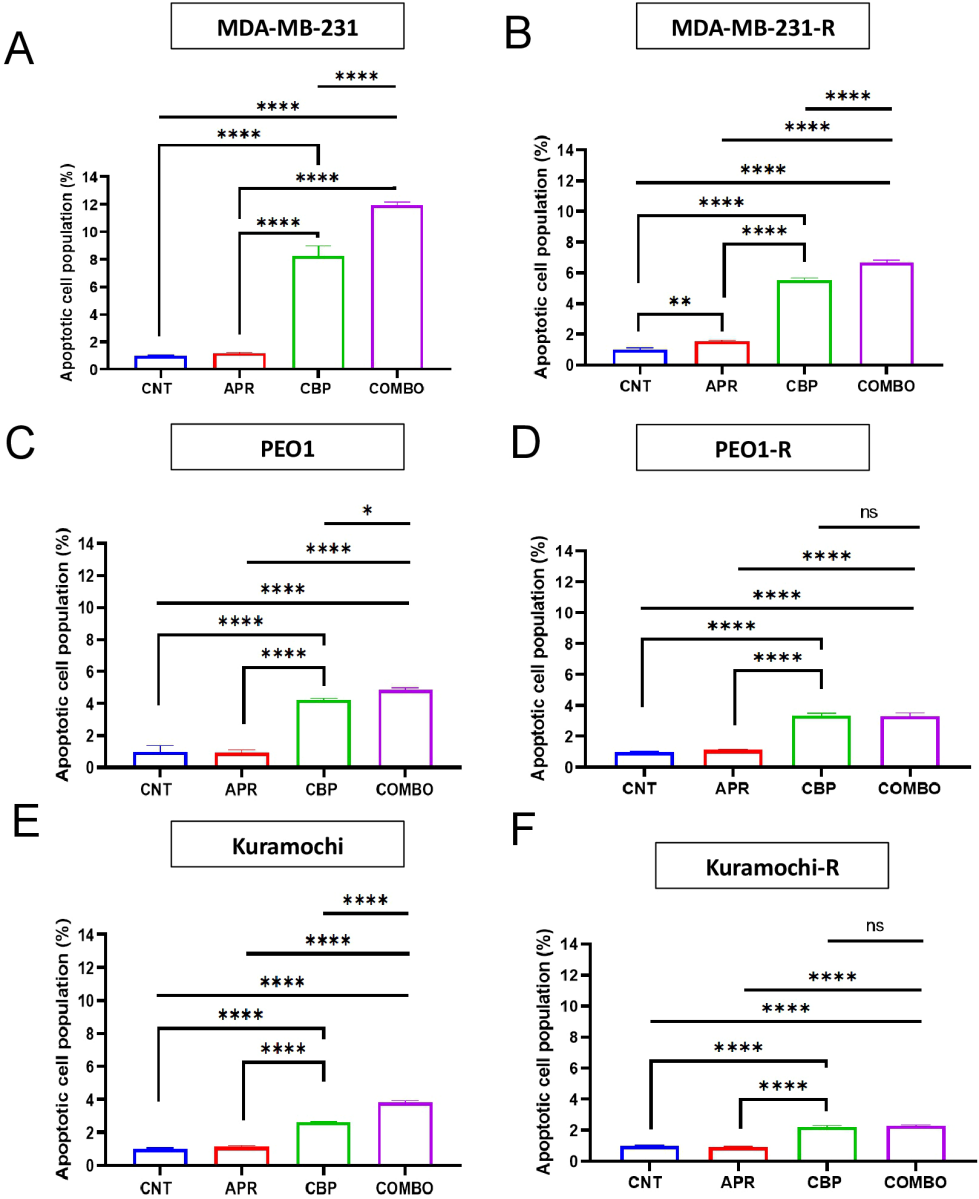
Eprenetapopt and carboplatin do not produce a consistent increase in apoptosis levels of TNBC and HGSOC olaparib-resistant cell lines. Bar graphs showing the percentage of apoptotic cells upon flow cytometry analyses in untreated cell lines (CNT) and after treating cells with the concentration corresponding to the IC_50_ value for their respective parental cell lines for each tretament: eprenetapopt monotherapy (APR) (MDA-MB-231 and MDA-MB-231-R 20 μM, Kuramochi and Kuramochi-R 20 μM and PEO1 and PEO1-R 15 μM), carboplatin monotherapy (CBP) (MDA-MB-231 and MDA-MB-231-R 400 μM, Kuramochi and Kuramochi-R 70 μM and PEO1 and PEO1-R 60 μM), and eprenetapopt + carboplatin combination treatment (COMBO) (combination of the previously cited doses of eprenetapopt and carboplatin for each cell line), for 72h in **(A)** MDA-MB-231, **(B)** MDA-MB-231-R, **(C)** PEO1, **(D)** PEO1-R, **(E)** Kuramochi and **(F)** Kuramochi-R cell lines. Bar graphs represent the mean ± SEM of n = 3 biological replicates. One-way ANOVA was used to assess differences between all conditions. *p< 0.05, **p< 0.01, ***p< 0.001, ****p< 0.0001.

We wondered if the combination of eprenetapopt + carboplatin increased apoptosis levels in the cell lines. To that, all cell lines were treated with eprenetapopt (APR), carboplatin (CBP) or the combination of both (COMBO) for 72h, stained with annexin V and propidium iodide and to analyze by flow cytometry the percentage of apoptotic cell population in each condition (see **Figure 4**). Flow cytometry analyses showed that eprenetapopt single agent (APR) induced no changes in the percentage of apoptotic cells compared control untreated cells (CNT) in all sensitive or resistant cells lines. Surprisingly a statistically significant increase in the apoptotic cell population when eprenetapopt (APR) was administered as a single agent compared to control condition (CNT) was observed in the MDA-MB-231-R cell line (**Figure 4B**).

The exposure to carboplatin single agent (CBP) induced a statistically significant and meaningful increase in the apoptosis of all sensitive and resistant cell lines compared to control untreated cells (CNT) and compared with eprenetapopt single agent (APR). The combination of eprenetapopt + carboplatin (COMBO) induced a statistically significant increase in the apoptosis levels compared to control untreated cells (CNT) as well as to the eprenetapopt single agent (APR), both in parental and resistant cell lines. However, when is compared to carboplatin single agent (CBP) treatment, a statistically significant increase in the apoptosis levels is observed in all parental cell lines (**Figures 4A, C, E**) and in the olaparib-resistant MDA-MB-231-R cell line (**Figure 4B**).

### Eprenetapopt + carboplatin treatment induces changes in the S or G2/M phases of the cell cycle in all TNBC and HGSOC cell lines

**Figure 5:**
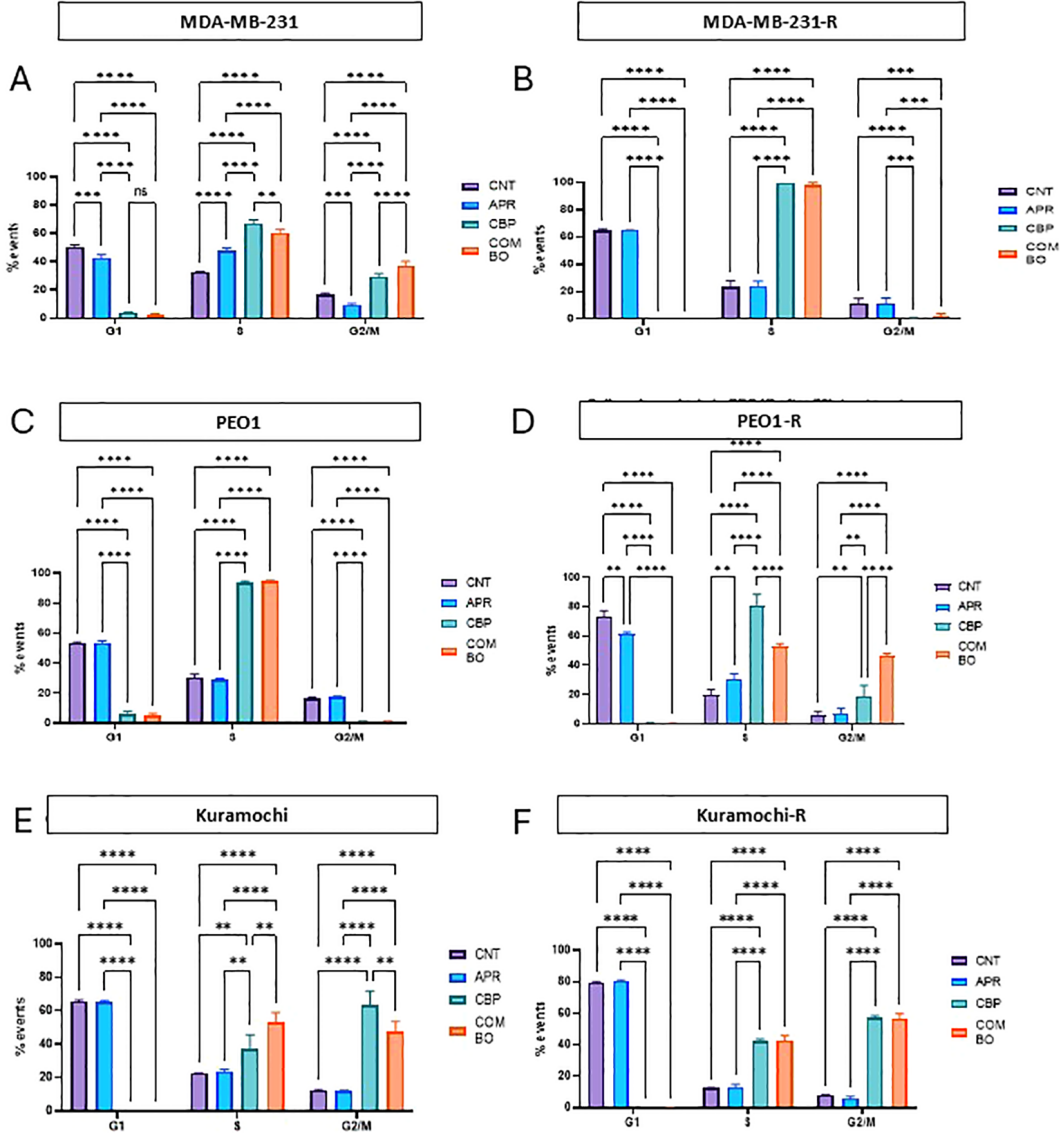
Eprenetapopt + carboplatin or carboplatin monotherapy induce an arrest in S or G2/M phase in the HGSOC and TNBC cell lines. Percentage of cell events detected by flow cytometry in untreated cell lines (CNT) and after treating cells with the concentration corresponding to the IC50 value for their respective parental cell lines for each tretament: eprenetapopt monotherapy (APR) (MDA-MB-231 and MDA-MB-231-R 20 μM, Kuramochi and Kuramochi-R 20 μM and PEO1 and PEO1-R 15 μM), carboplatin monotherapy (CBP) (MDA-MB-231 and MDA-MB-231-R 400 μM, Kuramochi and Kuramochi-R 70 μM and PEO1 and PEO1-R 60 μM), and eprenetapopt + carboplatin combination treatment (COMBO) (combination of the previously cited doses of eprenetapopt and carboplatin for each cell line), for 72h in **(A)** MDA-MB-231, **(B)** MDA-MB-231-R, **(C)** PEO1, **(D)** PEO1-R, **(E)** Kuramochi, **(F)** Kuramochi-R. Bar graphs represent the mean ± SEM of n = 3 biological replicates. One-way ANOVA was used to assess differences between all conditions. *p< 0.05, **p< 0.01, ***p< 0.001, ****p< 0.0001.

To further understand the mechanisms underlying the eprenetapopt + carboplatin combination, the effect on the cell cycle progression was assessed in all cell lines after exposure to eprenetapopt (APR), carboplatin (CBP) or the combination of both (COMBO) compared to control untreated cells (CNT) (**Figure 5**).

Overall, eprenetapopt single agent (APR) had a minimal impact on the cell cycle phase distribution across all lines. Carboplatin monotherapy (CBP), however, led to significant cell cycle modifications, with increased S-phase accumulation and a reduction in G1-phase cells when compared with untreated cells (CNT) and eprenetapopt in monotherapy (APR). This effect can be observed in all cell lines regardless of the presence of resistance to olaparib. In the MDA-MB-231, PEO1R, Kuramochi and Kuramochi-R cell lines, carboplatin (CBP) also increased the number of cells in G2/M.

The observed effect on the cell cycle of the combination treatment (COMBO) was very similar to that of carboplatin single agent (CBP), suggesting that the impact on cell cycle is mainly driven by carboplatin with a limited impact of the addition of eprenetapopt (APR). These findings suggest that while eprenetapopt (APR) enhances carboplatin (CBP) efficacy, its impact on cell cycle progression is minimal and further analyses would be needed to clarify which mechanisms underlying the synergistic effect observed in the eprenetapopt (APR) and carboplatin (CBP) combination treatment.

## Discussion

In the evolving landscape of cancer therapeutics, the integration of PARP inhibitors (PARPi) like olaparib into standard treatment regimens has become increasingly prevalent, particularly for patients with high-grade serous ovarian cancer (HGSOC) and triple-negative breast cancer (TNBC). The most frequent mechanism of PARPi resistance described to date is the reversion of the *BRCA* mutation, but the proportion of patients with this mechanism of resistance is low (17, 18). While the addition of eprenetapopt to carboplatin has previously demonstrated efficacy in overcoming carboplatin resistance in HGSOC patients not pre-treated with PARPi, the rising routine administration of olaparib necessitates an exploration of their combination effectiveness in the context of olaparib-resistant tumors. Addressing this gap is crucial, as resistance to PARPi can limit subsequent therapeutic options and adversely affect patient outcomes.

Our preclinical study successfully established olaparib-resistant TNBC and HGSOC cell line models through continuous exposure to olaparib, mirroring clinical scenarios where patients experience disease progression during PARPi treatment. Consistent with clinical observations (19) in patients, as demonstrated in PAOLA-1 clinical trial (20), our olaparib-resistant cell lines exhibited cross-resistance to platinum-based agents, evidenced by a significant increase in the IC_50_ of carboplatin. Notably, the method of inducing olaparib resistance varied among the cell lines; PEO1-R cells were subjected to high-dose olaparib pulses, whereas MDA-MB-231-R and Kuramochi-R cells underwent continuous low-dose exposure. This distinction is significant, as recent studies have demonstrated that different resistance induction methods can lead to divergent resistance mechanisms (21).

Given p53’s pivotal role in cell−cycle control and DNA−damage responses, we hypothesized that combining eprenetapopt—a compound that can restore wild−type–like p53 function—with carboplatin could yield synergistic cytotoxicity in TP53−mutant models. Our results support this hypothesis: the eprenetapopt–carboplatin combination demonstrated synergy across all parental and olaparib−resistant TP53−mutant HGSOC and TNBC cell lines analyzed, with Chou–Talalay combination index (CI) values consistently<1 at Fa = 0.25, 0.50, and 0.75. Notably, in MDA−MB−231−R and Kuramochi−R the CI at Fa = 0.5 was approximately 0.2, indicating strong synergy and, in these cases, exceeding that observed in their parental counterparts (≈0.4 and ≈0.6, respectively). These findings suggest that eprenetapopt plus carboplatin may represent a promising strategy to enhance cytotoxicity and address olaparib resistance in TP53−mutant HGSOC and TNBC.

We also evaluated the effect of the combination on apoptosis and cell−cycle distribution by flow cytometry. As expected, carboplatin monotherapy increased apoptosis across cell lines versus control, and the combination further increased apoptosis in the parental models; in resistant lines, a significant increment over carboplatin alone was observed in MDA−MB−231−R, whereas no consistent increase was detected in PEO1−R or Kuramochi−R. Regarding cell−cycle distribution, carboplatin drove S−phase accumulation and a reduction in G1−phase cells, and the combination produced a pattern largely similar to carboplatin monotherapy, suggesting that cell−cycle effects are predominantly carboplatin−driven in these settings. Altogether, these data indicate that while eprenetapopt enhances carboplatin cytotoxicity and yields synergy, the accompanying apoptosis and cell−cycle changes are context−dependent in resistant models and may reflect line−specific biology.

Clinically, these findings are most relevant to patients with TP53−mutant HGSOC or TNBC who progress on PARP inhibitors, regardless of BRCA1/2 status. A pragmatic translational path would enrich early−phase studies for tumors with NGS−confirmed TP53 mutation and prospectively explore pharmacodynamic markers of on−treatment DNA damage and/or p53 pathway engagement, acknowledging that such biomarkers were not assessed in the present work.

Limitations: This study was intentionally focused on efficacy readouts—dose–response viability, Chou–Talalay synergy, and flow−cytometry endpoints (apoptosis and cell−cycle)—and therefore did not include mechanistic assays, direct redox profiling, transcriptional readouts of p53 targets, or rescue experiments. Accordingly, our conclusions pertain to cytotoxicity and drug–drug interaction rather than the molecular mechanism of synergy. Dedicated mechanistic studies (e.g., glutathione/ROS assays, γH2AX immunoblotting or IHC, and p53 target gene expression) will be required in future work.

## Conclusions

Our findings indicate that, in this preclinical setting, the combination of carboplatin plus eprenetapopt is synergistic (CI<1) across MDA−MB−231, Kuramochi, and PEO1 models, including their olaparib−resistant derivatives. The combination enhanced cytotoxicity compared with single agents, while changes in apoptosis and cell−cycle distribution were context−dependent and largely driven by carboplatin. Although additional studies are required to define the molecular basis of this interaction and to refine patient−selection strategies, these data support further investigation of carboplatin plus eprenetapopt as a potential approach to address PARP inhibitor resistance in TP53−mutant TNBC and HGSOC.

## Data Availability

The raw data supporting the conclusions of this article will be made available by the authors, without undue reservation.
